# Automated radiosynthesis and in vivo evaluation of ^18^F-labeled analog of the photosensitizer ADPM06 for planning photodynamic therapy

**DOI:** 10.1186/s41181-023-00199-y

**Published:** 2023-07-17

**Authors:** Kazunori Kawamura, Tomoteru Yamasaki, Masayuki Fujinaga, Tomomi Kokufuta, Yiding Zhang, Wakana Mori, Yusuke Kurihara, Masanao Ogawa, Kaito Tsukagoe, Nobuki Nengaki, Ming-Rong Zhang

**Affiliations:** 1 Department of Advanced Nuclear Medicine Sciences, Institute for Quantum Medical Science, National Institutes for Quantum Science and Technology, 4-9-1 Anagawa, Inage-Ku, Chiba, 263-8555 Japan; 2 SHI Accelerator Service Ltd., 7-1-1 Nishigotanda, Shinagawa-Ku, Tokyo, 141-0032 Japan

**Keywords:** ^18^F, BF_2_-chelated tetraaryl-azadipyrromethenes, Photosensitizer, Photodynamic therapy

## Abstract

**Background:**

A family of BF_2_-chelated tetraaryl-azadipyrromethenes was developed as non-porphyrin photosensitizers for photodynamic therapy. Among the developed photosensitizers, ADPM06 exhibited excellent photochemical and photophysical properties. Molecular imaging is a useful tool for photodynamic therapy planning and monitoring. Radiolabeled photosensitizers can efficiently address photosensitizer biodistribution, providing helpful information for photodynamic therapy planning. To evaluate the biodistribution of ADPM06 and predict its pharmacokinetics on photodynamic therapy with light irradiation immediately after administration, we synthesized [^18^F]ADPM06 and evaluated its in vivo properties.

**Results:**

[^18^F]ADPM06 was automatically synthesized by Lewis acid-assisted isotopic ^18^F-^19^F exchange using ADPM06 and tin (IV) chloride at room temperature for 10 min. Radiolabeling was carried out using 0.4 μmol of ADPM06 and 200 μmol of tin (IV) chloride. The radiosynthesis time was approximately 60 min, and the radiochemical purity was > 95% at the end of the synthesis. The decay-corrected radiochemical yield from [^18^F]F^−^ at the start of synthesis was 13 ± 2.7% (*n* = 5). In the biodistribution study of male ddY mice, radioactivity levels in the heart, lungs, liver, pancreas, spleen, kidney, small intestine, muscle, and brain gradually decreased over 120 min after the initial uptake. The mean radioactivity level in the thighbone was the highest among all organs investigated and increased for 120 min after injection. Upon co-injection with ADPM06, the radioactivity levels in the blood and brain significantly increased, whereas those in the heart, lung, liver, pancreas, kidney, small intestine, muscle, and thighbone of male ddY mice were not affected. In the metabolite analysis of the plasma at 30 min post-injection in female BALB/c-*nu/nu* mice, the percentage of radioactivity corresponding to [^18^F]ADPM06 was 76.3 ± 1.6% (*n* = 3). In a positron emission tomography study using MDA-MB-231-HTB-26 tumor-bearing mice (female BALB/c-*nu/nu*), radioactivity accumulated in the bone at a relatively high level and in the tumor at a moderate level for 60 min after injection.

**Conclusions:**

We synthesized [^18^F]ADPM06 using an automated ^18^F-labeling synthesizer and evaluated the initial uptake and pharmacokinetics of ADPM06 using biodistribution of [^18^F]ADPM06 in mice to guide photodynamic therapy with light irradiation.

**Supplementary Information:**

The online version contains supplementary material available at 10.1186/s41181-023-00199-y.

## Background

Photodynamic therapy (PDT) is a minimally invasive therapeutic technique that combines visible or near-visible light with a photosensitizer (PS) for treating various types of cancers and other diseases (Abrahamse and Hamblin 2016; Kwiatkowski et al. 2018; Li et al. 2020). PDT is based on the ability of PSs to selectively accumulate in and kill tumor cells via the generation of reactive oxygen species (ROS) upon activation, guided by the light of a particular wavelength (Abrahamse and Hamblin 2016; Kwiatkowski et al. 2018; Li et al. 2020). Most PSs approved and used for the clinical treatment of solid tumors are porphyrin or phthalocyanine-based drugs (Li et al. 2020; Zhang et al. 2018). Other molecular structures, including synthetic dyes, have been developed as novel PSs for PDT (Abrahamse and Hamblin 2016; Zhang et al. 2018). Among the class of dyes used in sensor and fluorescent imaging, boron-fluorine derivatives (normally 4,4-difluoro-4-bora-3a,4a-diaza-s-indacene, BODIPY) have emerged as PSs for PDT (Awuah and You 2012; Kamkaew et al. 2013; Turksoy et al. 2019; Yao et al. 2013; Yu et al. 2020). BODIPYs have a large molar absorptive coefficient, extremely high chemical and photostability, and facile availability and can be structurally modified to achieve different properties (Awuah and You 2012; Kamkaew et al. 2013; Turksoy et al. 2019; Yao et al. 2013; Yu et al. 2020). In addition, BODIPYs have numerous characteristics that are ideal for use as PDT agents (low dark toxicity, high cellular uptake, high extinction coefficients, and low quantum yields for photobleaching). Hence, modifications that enable absorbance at long wavelengths are possible. Among the photosensitizing BODIPYs, ADPM06 was developed as part of a family of BF_2_-chelated tetraaryl-azadipyrromethenes (ADPMs) and displayed excellent photochemical and photophysical properties (Gorman et al. 2004). ADPM06 exhibited potent efficacy against a panel of cancer cell lines and effectively eradicated tumors in mouse xenograft models (Byrne et al. 2009; Gallagher et al. 2005; O’Connor et al. 2012). Furthermore, the in vivo efficacy of ADPM06 has been demonstrated in positron emission tomography (PET) studies using [^18^F]FDG and [^18^F]FLT (Byrne et al. 2009; Gallagher et al. 2005). Therefore, PET imaging may be useful for assessing PDT. In addition, the vascular targeting effect of ADPM06 was demonstrated by PET and MRI using a short drug-light interval (Byrne et al. 2009). The vascular-targeted PDT using ADPM06 was performed after an administration of 2 mg/kg of ADPM06 followed immediately by light irradiation (Byrne et al. 2009; O’Connor et al. 2012). The vascular-targeted PDT is effective since blood oxygen concentrations are high at vascular sites with high PS levels in blood shortly post-injection (Byrne et al. 2009). Therefore, the initial uptake of ADPM06 to blood is an important factor for vascular-targeted PDT. On the other hand, monitoring for PDT enables adaptation of delivery in case of inadequate response or prediction of response (Kharroubi Lakouas et al. 2017). Although optical imaging using photosensitizers can be considered as a biomarker for monitoring treatment response to superficial tumors, biodistribution of photosensitizers cannot be applicable to deep tumors or normal tissues. Consequently, PET using radiolabeled PS could allow its biodistribution to be traced, providing helpful information for planning PDT and predicting the pharmacokinetics of the PS (Kharroubi Lakouas et al. 2017). Moreover, PET monitoring before PDT may predict the side effects of the PS. In this study, we synthesized [^18^F]ADPM06 using an automated ^18^F-labeling synthesizer and evaluated its biodistribution in vivo to determine its use in monitoring or predicting the pharmacokinetics.

## Methods

### General

ADPM06 was purchased from BOC Sciences (Shirley, NY, USA). A 40 wt% aqueous solution of tetra-*n*-butylammonium bicarbonate (1.5 mol/L) was purchased from FutureChem (Seoul, Korea). The reagents and organic solvents were commercially obtained (Sigma-Aldrich, St. Louis, MO, USA; Kanto Chemical, Tokyo, Japan; Fujifilm Wako Pure Chemical, Osaka, Japan) and used without further purification.

Fluorine-18 as [^18^F]F^–^ was produced using a cyclotron (CYPRIS HM-18; Sumitomo Heavy Industries, Tokyo, Japan) and ^18^O(p, n)^18^F reaction in > 98 atom % ^18^O-enriched water (NUKEM Isotopes, Alzenau, Germany) bombarded with 18 MeV protons (14.2 MeV on target). [^18^F]ADPM06 was synthesized using a COSMiC-Compact Synthesizer (NMP Business Support Company, Sanda, Japan).

Semi-preparative high-performance liquid chromatography (HPLC) was performed using a radio-HPLC system (PU-2089 pump and UV-2070 detector, Jasco, Tokyo, Japan; NaI(Tl) scintillation detector, OKEN, Tokyo, Japan). Analytical HPLC was performed using a radio-HPLC system (515 pump and 2487 UV detector, Waters, Milford, MA, USA; NaI(Tl) scintillation detector; 925-SCNT ACE Mate single channel analyzer, ORTEC AMETEK, Oak Ridge, TN, USA; Model 51B51/2 scintillation detector, SCIONIX, Bunnik, Netherlands). For metabolite analysis, analytical HPLC was performed using a radio-HPLC system (PU-2089Plus pump and MD-2018Plus photodiode array detector, Jasco; bismuth germanate scintillation detector, S-2493Z, OKEN) (Takei et al. 2001).

The radioactivity of the samples for in vivo studies was determined using a 2480 Wizard 2 automatic gamma counter (PerkinElmer, Waltham, MA, USA). Unless otherwise stated, the radioactivity was determined using an IGC-7F Curiemeter (Hitachi, Tokyo, Japan).

### Radiosynthesis of [^18^F]ADPM06

**Fig. 1:**
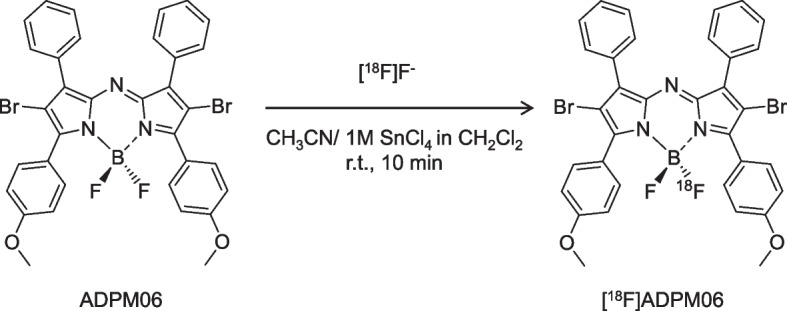
Radiosynthesis of [^18^F]ADPM06

[^18^F]ADPM06 was synthesized by Lewis acid-assisted isotopic ^18^F-^19^F exchange (Liu et al. 2013) with a slight modification using the ^18^F-labeling synthesizer, as described in the general section (Fig. 1). An aqueous [^18^F]F^–^ solution containing H_2_^18^O was passed through a Sep-Pak Accell Plus QMA Carbonate Plus Light cartridge (46 mg Waters) and washed with 1.5 mL of acetonitrile. [^18^F]F^–^ was eluted from the cartridge with a mixture of 1.5 mol/L tetrabutylammonium bicarbonate aqueous solution (0.15 mmol, 0.1 mL) and acetonitrile (0.9 mL). The mixture was introduced into a reaction vessel on the ^18^F-labeling synthesizer. The solution was concentrated by evaporation at 100 °C for 10 min under a nitrogen gas flow (100 mL/min). After cooling the reaction vessel, the mixture of ADPM06 (0.2–0.8 μmol) in acetonitrile (0.6 mL) and 1.0 mol/L tin(IV) chloride solution (SnCl_4_; 0.1–0.4 mmol, 0.1–0.4 mL) was added automatically in a syringe to the reaction vessel and agitated using a magnetic stirrer at room temperature for 10 min. After the reaction, the mixture was diluted with water for injection (0.2 mL) and transferred to an injector for semi-preparative HPLC, which was performed on the reaction mixture using the radio-HPLC system described in the general section. The semi-preparative HPLC conditions were as follows: InertSustainSwift C18 column (5 μm, 10 mm i.d. × 250 mm length; GL Sciences, Tokyo, Japan), acetonitrile/0.1% formic acid aqueous solution (95:5, v/v) as the mobile phase, a flow rate of 5 mL/min, and ultraviolet to visible light (UV–VIS) detection at 600 nm. The retention time of [^18^F]ADPM06 was ~ 10 min (Additional file 1: Fig. S1). The HPLC fractions of [^18^F]ADPM06 were collected in a flask to which Tween 80 (75 μL) in ethanol (0.3 mL) was added prior to radiosynthesis. The solution was subsequently evaporated, and the residue was dissolved in physiological saline.

The product was analyzed using HPLC described in the general section, and the analytical HPLC conditions were as follows: CAPCELL PAK C18 ACR column (3 μm, 4.6 mm i.d. × 100 mm length; Osaka Soda, Osaka, Japan), acetonitrile and 0.1% formic acid aqueous solution (90:10, v/v) as the mobile phase, a flow rate of 1 mL/min, and UV–Vis detection at 650 nm. The retention time for [^18^F]ADPM06 was 6.5 min (Additional file 1: Fig. S2).

### Animals

Male outbred laboratory mice (ddY, aged 7–8 weeks) and female inbred laboratory mice (BALB/c-*nu/nu*) were purchased from Japan SLC (Shizuoka, Japan).

### Biodistribution study in mice

[^18^F]ADPM06 (1.4 MBq/0.37 nmol) was intravenously injected into male ddY mice (aged 8 weeks; 36–40 g; *n* = 3 per group). Mice were sacrificed by cervical dislocation 5, 15, 30, 60, and 120 min after injection.

The effects of the co-injection with ADPM06 (2 mg/kg as the administration dose used for PDT) (Byrne et al. 2009; O’Connor et al. 2012) on tissue distribution were investigated. [^18^F]ADPM06 (1.2 MBq/0.23 nmol, 0.05 mL) and 2 mg/kg of ADPM06 in saline (0.1 mL) containing 0.75% Tween 80 and 3% ethanol were intravenously co-injected into male ddY mice (aged 8 weeks; 35–38 g; *n* = 4). The mice were sacrificed by cervical dislocation 30 min after injection.

Blood samples were collected by heart puncture. Heart, lung, liver, pancreas, spleen, kidney, small intestine, muscle, brain, and thighbone were dissected and weighed. Radioactivity in the samples was measured using an automatic gamma counter as described in the general section. The distribution of radioactivity was expressed as a percentage of the injected dose per gram of tissue (% ID/g, tissue radioactivity/gram tissue/injected radioactivity × 100).

### Metabolite analysis of plasma in mice

[^18^F]ADPM06 (29 MBq/14 nmol) was intravenously injected into female BALB/c-*nu/nu* mice (aged 16 weeks; 18–20 g; *n* = 3). The mice were sacrificed by cervical dislocation 30 min after injection. Blood samples were collected via heart puncture in heparinized syringes. The samples were centrifuged at 10,000×*g* (Model 5500, KUBOTA, Tokyo, Japan) for 3 min at 4 °C to obtain plasma (0.1–0.2 mL). Plasma samples were added to an equivalent volume of 20% trichloroacetic acid in acetonitrile on ice, vortexed, and centrifuged at 20,000×*g* for 2 min; subsequently, the supernatant was collected. The precipitate was added to 0.5 mL of 20% trichloroacetic acid in acetonitrile on ice, and the supernatant was collected following the same treatment as plasma samples. The supernatants were mixed, centrifuged at 20,000×*g* for 2 min, and loaded into the HPLC injector loop. HPLC analysis was performed using the radio-HPLC system for metabolite analysis described in the general section, as follows: XSelect CSH C18 column (5 μm, 10 mm i.d. × 100 mm, Waters), a mixture of acetonitrile and 0.1% formic acid solution (90:10, v/v) as the mobile phase, 4.0 mL/min flow rate, and 650 nm for UV–Vis detection. The retention time of [^11^C]ADPM06 was approximately 7.3 min (Additional file 1: Fig. S3). Radioactivity in the supernatants, precipitates, and HPLC waste was measured using an automatic gamma counter, as described in the general section. The recovery of radioactivity from plasma into supernatant for deproteinized-treatment was 98.4 ± 0.1% (*n* = 3).

### PET study in a tumor-bearing mouse

Female BALB/c-*nu/nu* mice were anesthetized using isoflurane, and MDA-MB-231-HTB-26 cells (ATCC, Manassas, VA, USA) were injected subcutaneously into the right lower limb according to a previously described method (O’Connor et al. 2012). The mouse tumors grew until they reached approximately 300 mm^2^. The mouse was secured in a custom-designed chamber and placed in a small-animal PET scanner (Inveon; Siemens Medical Solutions, Knoxville, TN, USA). The body temperature was maintained using a 40 °C water circulation system (T/Pump TP401; Gaymar Industries, Orchard Park, NY, USA). A bolus of [^18^F]ADPM06 (3.3 MBq/1.3 nmol) was injected via a catheter inserted into the tail vein. Dynamic emission scans in three-dimensional list mode were performed for 60 min. Immediately after the PET scan, the mice were sacrificed by cervical dislocation, and the tissue or tumor dissection was performed according to the method described in the section on biodistribution studies in mice. For the PET study, the decay-corrected radioactivity was expressed as a standardized uptake value (SUV; tissue radioactivity/tissue volume/injected radioactivity × total body weight).

### Statistical analysis

Quantitative data are expressed as mean ± standard deviation (SD). Differences between control mice and mice co-injected with ADPM06 were examined using non-parametric Mann–Whitney test and considered significant at *P* < 0.05. Data were analyzed using the OriginPro 2023 software package (OriginLab, Northampton, MA, USA).

## Results

### Radiosynthesis

**Fig. 2:**
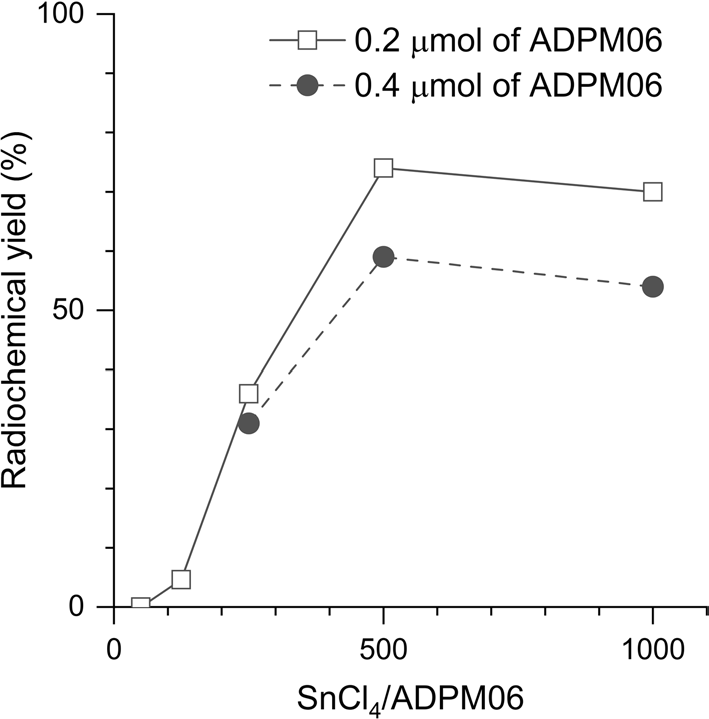
Radiochemical yields of [^18^F]ADPM06 with varying concentration ratios of SnCl_4_ to ADPM06. The decay-corrected radiochemical yield was based on HPLC analysis of the reaction mixture

To efficiently synthesize [^18^F]ADPM06, we manually optimized the one-pot ^18^F-labeling of [^18^F]ADPM06 using [^18^F]tetrabutylammonium fluoride (TBAF), which was prepared in the ^18^F^−^ elution step using an automated ^18^F-labeling synthesizer (Fig. 1). When 0.2 μmol of ADPM06 was used as a precursor, the decay-corrected radiochemical yield (RCY) of [^18^F]ADPM06 from [^18^F]TBAF at the start of synthesis (SOS) increased to 74% (based on HPLC analysis of the crude product) on increasing the concentration ratio of SnCl_4_/ADPM06 from 50:1 to 500:1 but did not increase with further increase in the ratio of SnCl_4_/ADPM06 (Fig. 2). When 0.4 μmol of ADPM06 was used, the RCY of [^18^F]ADPM06 from [^18^F]TBAF at the SOS increased to 59% (based on HPLC analysis of the crude product) on increasing the concentration ratio of SnCl_4_/ADPM06 to 500:1 (Fig. 2).

Under the optimized reaction conditions, we performed the automated synthesis of [^18^F]ADPM06 using an ^18^F-labeling synthesizer. Under an SnCl_4_/ADPM06 concentration ratio of 500:1, the RCYs at the SOS were 4.4%, 13%, and 14% when 0.2, 0.4, and 0.8 μmol of ADPM06 were used as precursors for radiolabeling, respectively (Table 1). Above the 500:1 ratio of SnCl_4_/ADPM06, the RCY at the SOS increased when 0.2 and 0.4 μmol of ADPM06 were used as precursors, although radiochemical purity was decreased (Table 1). The radiosynthesis time was approximately 60 min, and the radiochemical purity was > 95% (Additional file 1: Fig. S2) for 1 h at the end of synthesis. The molar activity using 0.4 μmol of ADPM06 for the reaction at the end of synthesis was 4.0 ± 0.6 GBq/μmol (*n* = 5).

**Table 1:** Radiosynthesis of [^18^F]ADPM06 using the automated ^18^F-labeling synthesizer

ADPM06 (μmol)	SnCl_4_ (μmol)	SnCl_4_/ADPM06	RCY (%)	RCP (%)
0.2	100	500:1	4.4	> 99
0.2	200	1000:1	11 ± 7.9^a^	> 98
0.4	200	500:1	13 ± 2.7^b^	> 99
0.4	300	750:1	37	> 95
0.8	400	500:1	14	> 99

^a^Mean ± SD (*n* = 3)

^b^Mean ± SD (*n* = 5)

### Biodistribution study in mice

**Fig. 3:**
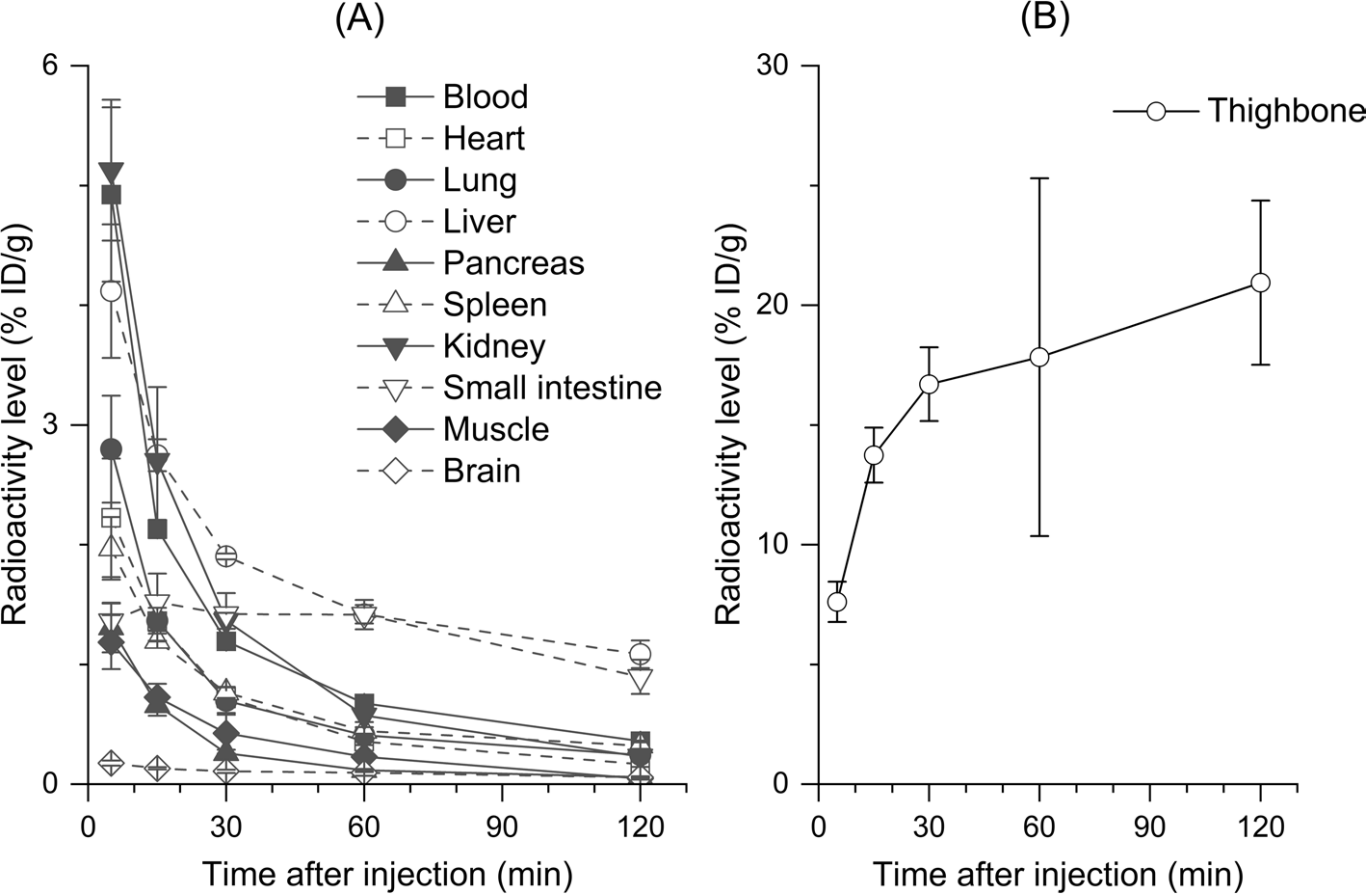
Biodistribution of radioactivity in various organs except for thighbone (**A**) and thighbone (**B**) of mice at 5, 15, 30, 60, and 120 min after [^18^F]ADPM06 injection. [^18^F]ADPM06 (1.4 MBq/0.37 nmol) was intravenously injected into male ddY mice (*n* = 3 per group). The radioactivity level is expressed as % ID/g

The results of radioactivity biodistribution following the injection of [^18^F]ADPM06 in mice are shown in Fig. 3. After injection of [^18^F]ADPM06, the mean radioactivity levels in the blood, heart, lung, liver, pancreas, spleen, kidney, muscle, and brain gradually decreased for 120 min after the initial uptake (Fig. 3A). The mean radioactivity level in the small intestine increased for 15 min post-injection and decreased slightly 30 min following injection (Fig. 3A). To study the effects of ^18^F-defluorination, we investigated thighbone uptake after the injection of [^18^F]ADPM06. The mean radioactivity level in the thighbone was the highest among all the organs investigated and increased for 120 min after injection (Fig. 3B).

**Fig. 4:**
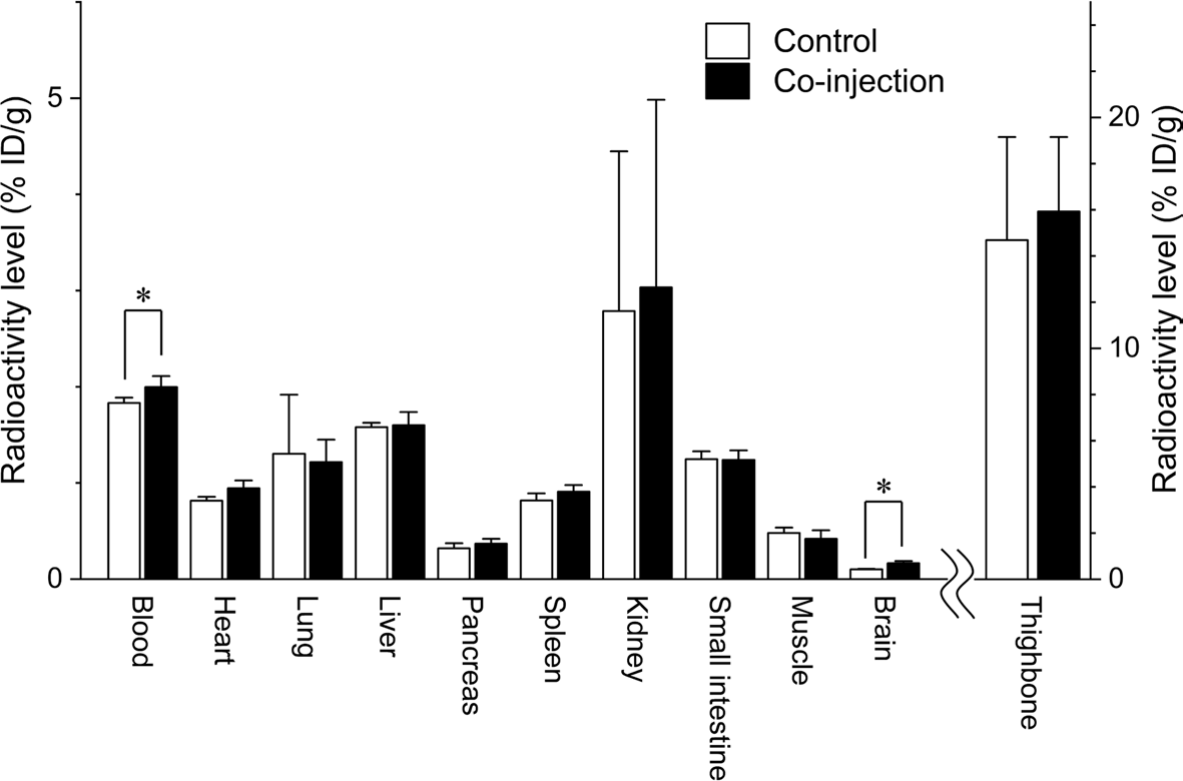
Radioactivity levels following co-injection with ADPM06 at 30 min after [^18^F]ADPM06 injection. [^18^F]ADPM06 (1.2 MBq/0.23 nmol) and ADPM06 (2 mg/kg) were intravenously co-injected into male ddY mice (*n* = 4). *P < 0.05 (non-parametric Mann–Whitney test, compared with control)

The effects of co-injection with ADPM06 (2 mg/kg as the administration dose used for PDT) (Byrne et al. 2009; O’Connor et al. 2012) on radioactivity biodistribution after the injection of [^18^F]ADPM06 in mice are shown in Fig. 4. Upon co-injection with ADPM06, the radioactivity levels in the blood and brain significantly increased (110% and 160%, respectively), whereas those in the heart, lung, liver, pancreas, kidney, small intestine, muscle, and thighbone were not affected.

### Metabolite study of plasma in mice

The percentage of radioactivity in the plasma of mice at 30 min after the injection of [^18^F]ADPM06 was investigated. The percentage of radioactivity corresponding to [^18^F]ADPM06 peak form HPLC was 95.6 ± 0.5% (*n *= 3; Additional file 1: Fig. S3). However, the recovery of radioactivity from HPLC waste was 79.8 ± 1.3% (*n *= 3), as measured by quantitative radio-HPLC analysis. From the result of the corrected radioactivity corresponding to absorbed ^18^F^-^ on the HPLC column, the percentage of radioactivity corresponding to [^18^F]ADPM06 in the plasma of mice was 76.3 ± 1.6% (*n *= 3).

### PET study in a tumor-bearing mouse

**Fig. 5:**
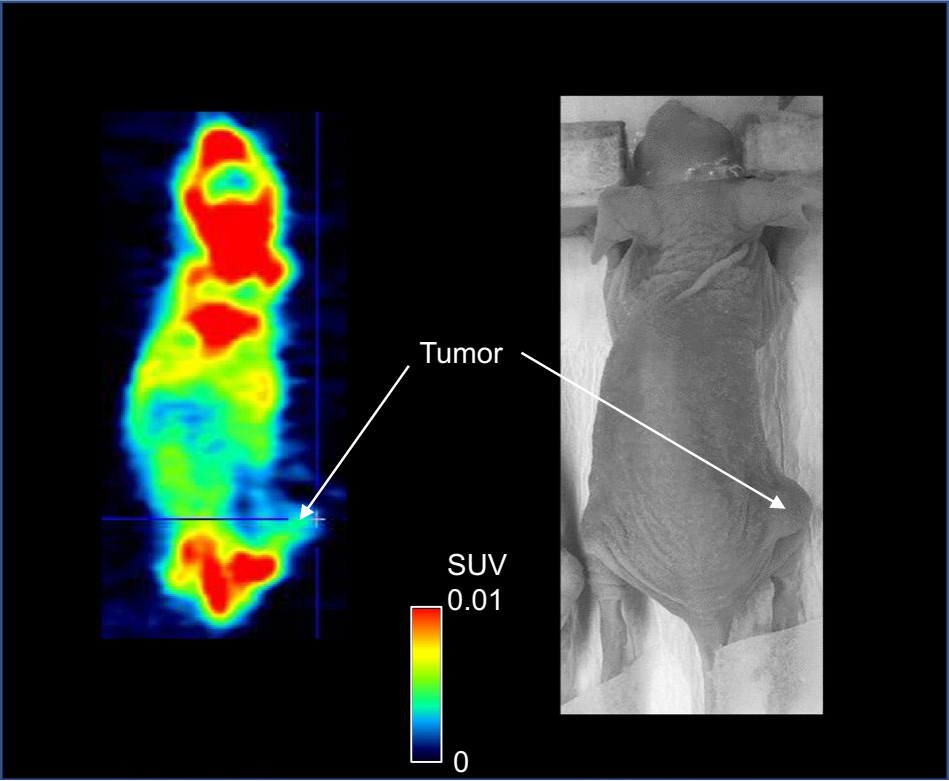
PET image of an MDA-MB-231-HTB-26 tumor-bearing mouse (female BALB/c-*nu/nu*) injected with [^18^F]ADPM06 (3.3 MBq/1.3 nmol). PET images were acquired for 60 min following injection. Mice were anesthetized with isoflurane and placed in the prone position on the bed of the scanner. The scale of radioactivity is expressed as SUV

**Fig. 6:**
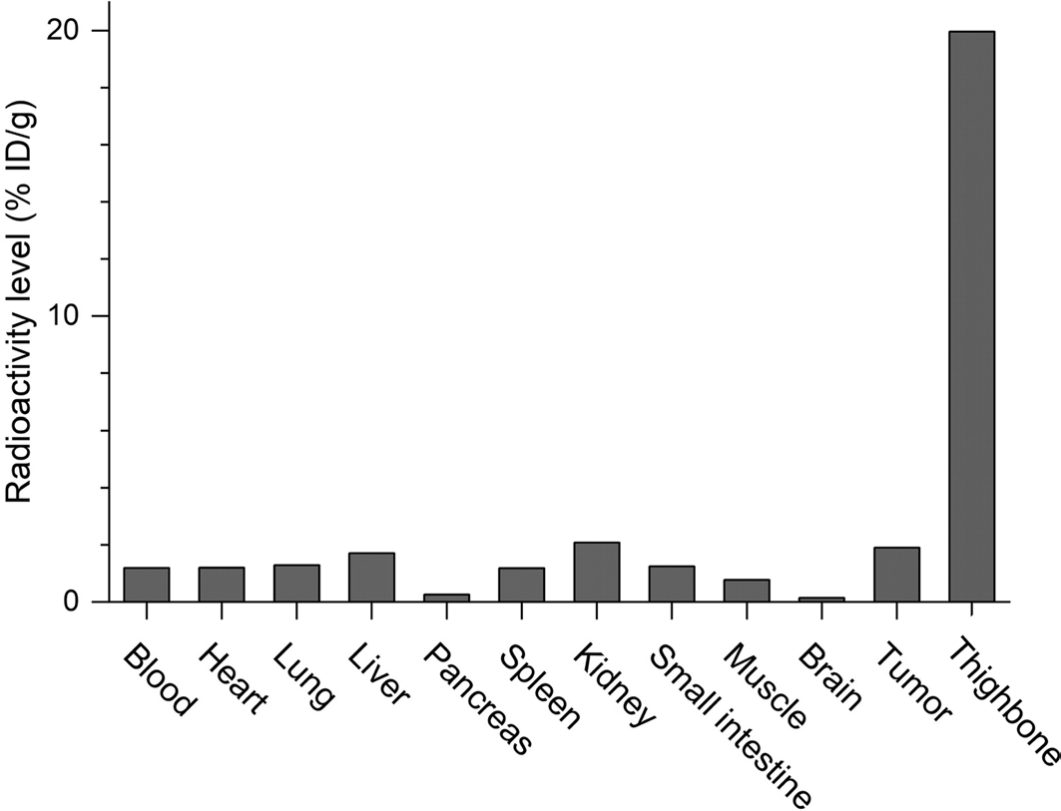
Analysis of biodistribution of radioactivity immediately after the 60 min PET scan in an MDA-MB-231-HTB-26 tumor-bearing mouse (female BALB/c-*nu/nu*) injected with [^18^F]ADPM06. [^18^F]ADPM06 (3.3 MBq/1.3 nmol) was intravenously injected into the tumor-bearing mouse. The radioactivity level is expressed as % ID/g

Figure 5 shows a PET image of an MDA-MB-231-HTB-26 tumor-bearing mouse acquired by scanning for 60 min after [^18^F]ADPM06 injection. A relatively high accumulation of radioactivity was observed in the bone (Fig. 5). Radioactivity after the injection of [^18^F]ADPM06 accumulated to a moderate level in the tumor. (Fig. 5). A biodistribution study immediately after a 60 min PET scan is shown in Fig. 6. The radioactivity level in the thighbone was the highest among all the investigated tissues, followed by kidney, tumor, liver, lung, blood, heart, spleen, and small intestine at a moderate level (Fig. 6). At 60 min after the injection, radioactivity distributions and levels in female BALB/c-*nu/nu* mice as mentioned above were similar to those in male ddY mice.

## Discussion

We successfully synthesized [^18^F]ADPM06 using Lewis-acid-assisted isotopic ^18^F-^19^F exchange. To improve the radiochemical yield of [^18^F]ADPM06 by isotopic ^18^F-^19^F exchange, we increased the concentration of SnCl_4_ because SnCl_4_ is remarkably efficient at promoting ^18^F-^19^F isotopic exchange, even at room temperature (Liu et al. 2013). In the manual radiosynthesis, the radiochemical yield using 0.2 μmol of ADPM06 as a precursor was higher than that using 0.4 μmol of ADPM06. However, in the automated radiosynthesis, the radiochemical yield using 0.2 μmol of ADPM06 was lower than that using 0.4 μmol of ADPM06. As the difference between manual and automated radiosynthesis is the time for mixing ADPM06 with SnCl_4_ solution until reaction, the reaction efficiency may be decreased when a relatively low concentration of ADPM06 and SnCl_4_ was used. Therefore, we chose 0.4 μmol of ADPM06 for the automated radiosynthesis of [^18^F]ADPM06. Among the investigated ratios of SnCl_4_ to ADPM06 ranging from 50:1 to 1000:1, the RCY of [^18^F]ADPM06 from [^18^F]TBAF at the SOS was the highest when an SnCl_4_/ADPM06 concentration ratio of 500:1 was used. However, we observed a decrease in the yield at a very high concentration ratio of SnCl_4_/ADPM06 (≥ 1000-fold that of SnCl_4_ to ADPM06). This phenomenon may be explained by the sequestration of fluoride from ^18^F-labeled BODIPYs by excess tin reagent (Liu et al. 2013). Under optimized conditions, we performed automated and reproducible radiosynthesis of [^18^F]ADPM06 with high radiochemical purity (> 99%; Additional file 1: Fig. S2) and appropriate radiochemical yield for in vivo experiments (13%) using 0.4 μmol of ADPM06 and 200 μmol of SnCl_4_.

In biodistribution studies, the radioactivity levels in the lung, heart, spleen, and kidney showed time-activity courses similar to those in previous biodistribution studies using optical imaging (Byrne et al. 2009). In contrast, radioactivity in the liver showed relatively fast accumulation for 15 min after the initial uptake, whereas optical imaging showed slow accumulation in the liver for 30 min after the initial uptake (Byrne et al. 2009). This result may be explained by the existence of ^18^F-labeled radiometabolites. The radioactivity level in the thighbone was high and gradually increased for 120 min after the injection. [^18^F]ADPM06 may be gradually metabolized and absorbed by the bone via ^18^F-defluorination. As seen from the result of the metabolite analysis in the plasma of mice, the radioactivity corresponding to ^18^F^−^ absorbed on the HPLC column accounted for approximately at 20% in the plasma for 30 min after injection of [^18^F]ADPM06 because the silica-based HPLC column retained ^18^F^−^ in low pH (≤ 3) conditions (Ory et al. 2015). Thus, [^18^F]ADPM06 possibly gradually metabolized to ^18^F^−^ in vivo. The accumulation of radioactivity in bones has previously been ascribed to the metabolic instability of the borane-fluorine bond of [^18^F]BODIPY derivatives (Paulus et al. 2015). Different BODIPY core structures exhibit different metabolic half-lives (Paulus et al. 2015).

Additionally, we investigated the effects of co-injection with ADPM06 (2 mg/kg as the administration dose used for PDT) (Byrne et al. 2009; O’Connor et al. 2012). The radioactivity levels at 30 min after the co-injection of [^18^F]ADPM06 with ADPM06 were not altered in the heart, lungs, liver, pancreas, spleen, kidney, small intestine, muscle, or thighbone, although that in the brain was increased by co-injection with ADPM06 because of increased radioactivity levels in the blood. Based on these results, the pharmacokinetics of ADPM06 while performing PDT with light irradiation in individual animals and humans should be evaluated in the PET study using [^18^F]ADPM06 to predict the side effects on normal tissues and organs due to light toxicity. For example, this study showed high ^18^F^−^ accumulation in thighbone, indicating that in vivo defluorination of ADPM06 could decrease the therapeutic efficiency of PDT. In future, development of more stable ADPM06 analogs in vivo is required, and PET studies with its corresponding radiolabeled analogs should be conducted to evaluate the efficiency of PDT.

## Conclusions

We conducted the automatic synthesis of [^18^F]ADPM06 by Lewis acid-assisted isotopic ^18^F-^19^F exchange using an ^18^F-labeling synthesizer. In addition, to guide PDT with light irradiation, we evaluated the initial uptake using biodistribution studies of [^18^F]ADPM06 in mice and in vivo properties of [^18^F]ADPM06 using PET in tumor-bearing mice, and analyzed radiolabeled metabolites in mice.

## Supplementary Information

**Additional file 1**. Supplementary information on chromatograms of typical semi-preparative HPLC, analytical HPLC, and radio-HPLC for metabolite analysis.

## Data Availability

Data are provided in the article and supplementary information.

## Abbreviations

PDT: Photodynamic therapy
PS: Photosensitizer
ROS: Reactive oxygen species
BODIPY: 4,4-Difluoro-4-bora-3a,4a-diaza-s-indacene
ADPM: BF_2_-chelated tetraaryl-azadipyrromethene
PET: Positron emission tomography
HPLC: High-performance liquid chromatography
UV–VIS: Ultraviolet to visible light
%ID/g: Percentage of the injected dose per gram of tissue
SUV: Standardized uptake value
SD: Standard deviation
TBAF: Tetrabutylammonium fluoride
RCY: Decay-corrected radiochemical yield
SOS: Start of synthesis

## References

[CR1] Abrahamse H, Hamblin MR (2016). New photosensitizers for photodynamic therapy. Biochem J.

[CR2] Awuah SG, You Y (2012). Boron dipyrromethene (BODIPY)-based photosensitizers for photodynamic therapy. RSC Adv.

[CR3] Byrne AT, O’Connor AE, Hall M, Murtagh J, O’Neill K, Curran KM (2009). Vascular-targeted photodynamic therapy with BF_2_-chelated tetraaryl-azadipyrromethene agents: a multi-modality molecular imaging approach to therapeutic assessment. Br J Cancer.

[CR4] Gallagher WM, Allen LT, O’Shea C, Kenna T, Hall M, Gorman A (2005). A potent nonporphyrin class of photodynamic therapeutic agent: cellular localisation, cytotoxic potential and influence of hypoxia. Br J Cancer.

[CR5] Gorman A, Killoran J, O’Shea C, Kenna T, Gallagher WM, O’Shea DF (2004). In vitro demonstration of the heavy-atom effect for photodynamic therapy. J Am Chem Soc.

[CR6] Kamkaew A, Lim SH, Lee HB, Kiew LV, Chung LY, Burgess K (2013). BODIPY dyes in photodynamic therapy. Chem Soc Rev.

[CR7] Kharroubi Lakouas D, Huglo D, Mordon S, Vermandel M (2017). Nuclear medicine for photodynamic therapy in cancer: planning, monitoring and nuclear PDT. Photodiagnosis Photodyn Ther.

[CR8] Kwiatkowski S, Knap B, Przystupski D, Saczko J, Kędzierska E, Knap-Czop K (2018). Photodynamic therapy-mechanisms, photosensitizers and combinations. Biomed Pharmacother.

[CR9] Li X, Lovell JF, Yoon J, Chen X (2020). Clinical development and potential of photothermal and photodynamic therapies for cancer. Nat Rev Clin Oncol.

[CR10] Liu S, Lin TP, Li D, Leamer L, Shan H, Li Z (2013). Lewis acid-assisted isotopic ^18^F–^19^F exchange in BODIPY dyes: facile generation of positron emission tomography/fluorescence dual modality agents for tumor imaging. Theranostics.

[CR11] O’Connor AE, Mc Gee MM, Likar Y, Ponomarev V, Callanan JJ, O’Shea DF (2012). Mechanism of cell death mediated by a BF_2_-chelated tetraaryl-azadipyrromethene photodynamic therapeutic: dissection of the apoptotic pathway in vitro and in vivo. Int J Cancer.

[CR12] Ory D, Van den Brande J, de Groot T, Serdons K, Bex M, Declercq L (2015). Retention of [^18^F]fluoride on reversed phase HPLC columns. J Pharm Biomed Anal.

[CR13] Paulus A, Desai P, Carney B, Carlucci G, Reiner T, Brand C (2015). Development of a clickable bimodal fluorescent/PET probe for in vivo imaging. EJNMMI Res.

[CR14] Takei M, Kida T, Suzuki K (2001). Sensitive measurement of positron emitters eluted from HPLC. Appl Radiat Isot.

[CR15] Turksoy A, Yildiz D, Akkaya EU (2019). Photosensitization and controlled photosensitization with BODIPY dyes. Coord Chem Rev.

[CR16] Yao L, Xiao S, Dan F (2013). Boron-fluorine photosensitizers for photodynamic therapy. J Chem.

[CR17] Yu Z, Zhou J, Ji X, Lin G, Xu S, Dong X (2020). Discovery of a monoiodo aza-BODIPY near-infrared photosensitizer: in vitro and in vivo evaluation for photodynamic therapy. J Med Chem.

[CR18] Zhang J, Jiang C, Figueiró Longo JP, Azevedo RB, Zhang H, Muehlmann LA (2018). An updated overview on the development of new photosensitizers for anticancer photodynamic therapy. Acta Pharm Sin B.

