# Supplementary material for: Automated radiosynthesis and in vivo evaluation of 18F-labeled analog of the photosensitizer ADPM06 for planning photodynamic therapy
Source: EJNMMI Radiopharm Chem. 2023 Jul 17;8:14. doi: 10.1186/s41181-023-00199-y (PMC10352184; doi:10.1186/s41181-023-00199-y)
Supplement: Supplementary file 1 — Additional file 1. Supplementary information on chromatograms of typical semi-preparative HPLC, analytical HPLC, and radio-HPLC for metabolite analysis. [file 41181_2023_199_MOESM1_ESM.docx]

**Additional file 1 of “****Automated radiosynthesis and *in vivo* evaluation of ^18^F-labeled analog of the photosensitizer ADPM06 for planning photodynamic therapy”**

**Supplemtary information**

**Fig. S1**

Fig. S1. Typical semi-preparative HPLC choromatogram of the [^18^F]ADPM06 fraction using a radio-HPLC. The semi-preparative HPLC conditions were as follows: InertSustainSwift C18 column (5 μm, 10 mm i.d. × 250 mm length), acetonitrile/0.1% formic acid aqueous solution (95:5, vol./vol.) as the mobile phase, a flow rate of 5 mL/min, UV-VIS detection at 600 nm, and NaI(Tl) scintilattion detection.

**Fig. S2**

Fig. S2. Typical analytical HPLC choromatogram of the [^18^F]ADPM06 injection using a radio-HPLC. The analytical HPLC conditions were as follows: CAPCELL PAK C18 ACR column (3 μm, 4.6 mm i.d. × 100 mm length), acetonitrile and 0.1% formic acid aqueous solution (90:10, vol./vol.) as the mobile phase, a flow rate of 1 mL/min, and UV-VIS detection at 650 nm.

**Fig. S3**

Fig. S3. Typical radio-HPLC chromatogram for metabolite analysis of plasma in BALB/c-*nu/nu* mice at 30 min after the [^18^F]ADPM06 injection. The HPLC condition were as follows: XSelect CSH C18 column (5 μm, 10 mm i.d. × 100 mm), a mixture of acetonitrile and 0.1% formic acid solution (90:10, vol./vol.) as a mobile phase, 4.0 mL/min flow rate, and bismuth germanate scintilation detection.
